# Accelerated Uptake of CO_2_ Catalyzed by Immobilized Thermophilic Carbonic Anhydrase on Dispersed Aminated Mesoporous Silica

**DOI:** 10.1021/acsami.5c08889

**Published:** 2025-10-28

**Authors:** Maja-Stina Svanberg Frisinger, Didem Mimiroglu, Latif Ullah, Swati Verma, Mats Martinelle, Per Berglund, Niklas Hedin

**Affiliations:** † Department of Chemistry, 7675 Stockholm University, SE-10691 Stockholm, Sweden; ‡ Department of Biochemistry, Faculty of Science, Sivas Cumhuriyet University, 58140 Sivas, Turkey; § Department of Industrial Biotechnology, 7655 KTH Royal Institute of Technology, AlbaNova University Center, SE-106 91 Stockholm, Sweden

**Keywords:** Biocatalysis, CO_2_ capture, enzyme immobilization, aminated silica

## Abstract

Efficient and durable biocatalysts are important for sustainable CO_2_ capture technologies, but enzyme stability often limits their use under harsh process conditions. Here, we evaluate carbonic anhydrases (CAs) adsorbed onto aminated mesoporous SBA-15 as biocatalysts for CO_2_ capture under the hypothesis of adsorption-induced thermal stabilization. Carbonic anhydrase from the thermophilic bacterium *Persephonella marina* (pmCA) and commercial bovine erythrocyte carbonic anhydrase (bCA) were used. Enzyme adsorption isotherms for pmCA and bCA onto the aminated SBA-15 were established, along with desorption tests. Adsorbed and free pmCA and bCA were incubated at 40–90 °C for 14 d. The structural integrity and possibility of amine leaching of the incubated (90°, 14 d) aminated SBA-15 were analyzed by X-ray diffraction (XRD) and NMR spectroscopy. The reaction product speciation in CO_2_-loaded catalyzed and uncatalyzed dispersions was monitored using infrared (IR) spectroscopy. The maximum enzyme adsorption capacities were established to be 1.4 ± 0.2 g pmCA·g-aminated SBA-15^–1^ and 2.1 ± 0.5 g bCA·g-aminated SBA-15^–1^, with no detectable desorption. Adsorbed pmCA and bCA maintained high activity for 14 d at 40–65 °C and for 4 d at 90 °C, whereas free enzymes lost activity within 4 d at all temperatures. The XRD patterns of the heat-treated (90 °C, 14 d) aminated SBA-15 indicated a full collapse of the mesostructure. IR spectroscopy confirmed enhanced HCO_3_^–^ formation in the presence of immobilized CA. Overall, enzyme adsorption onto the aminated SBA-15 significantly improved the thermal stability and activity of pmCA and bCA compared to the free enzymes, demonstrating the potential of adsorbed CAs for biocatalysis.

## Graphical abstract

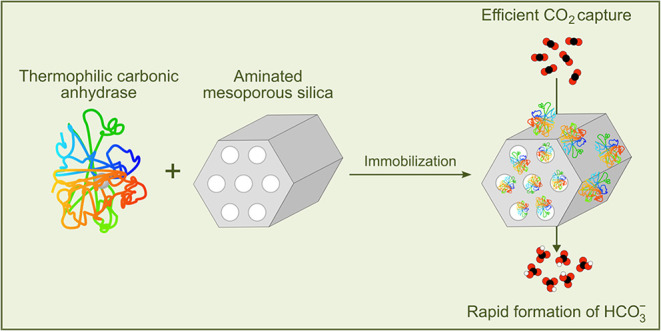

## 1 Introduction

The separation of CO_2_ from biogenic industrial flue gases, bioenergy carbon capture and storage (BECCS), has gained attention as a means of simultaneously generating negative CO_2_ emissions and producing heat and power.^1,2^ The separation process is based on the chemical absorption of CO_2_ into an aqueous basic scrubbing fluid, from which CO_2_ is released in the next stage of thermal swing operation. Catalysts or rate promoters are typically added to scrubbing fluids to enhance reaction rates and capacities. Traditional catalysts include inorganic substances such as arsenites, boric acid, and vanadates, and organic substances, such as alkanolamines and amino acids.^3–7^ Carbonic anhydrase (CA) catalyzes the hydration of CO_2_ into HCO_3_^–^ and has been suggested as an environmentally friendly and efficient replacement for traditional catalysts due to its high hydration rates, with a turn over frequency (TOF) up to 10^6^ s^–1^.^8^

The use of CA in biocatalysis and CO_2_ separation processes must involve care, particularly with respect to the harsh conditions to which CA might be subjected. Temperature, pH, and ionic strength change rapidly throughout the cyclic absorption–desorption process, which may lead to irreversible activity loss due to denaturation unless enhanced stability is ensured. There are numerous ways to increase the stability of enzymes, including the use of thermophilic CA variants and immobilization on a solid support.^9–16^ Such adsorption-induced stability is linked to the shielding of the hydrophobic parts of the enzyme and the reduction of entropy connected to the polypeptide chain upon adsorption; hence, the rate of denaturation may also be reduced.^17,18^

The hydration of CO_2_ by CA-s has been shown to occur in a four-stage sequence, involving the conversion of CO_2_ to HCO_3_^–^ by a zinc-coordinated hydroxyl ion (Scheme 1a). This is followed by a series of proton transfers that regenerate the original enzyme structure, where regeneration of the active site by diffusion is the rate-limiting step in the hydration of CO_2_. The CO_2_ activity of CA is often difficult to measure due to the lack of precise and accurate methods, particularly at higher temperatures. Thus, CA activity is often inferred from its esterase activity because of the similar reaction mechanisms at the active site. Crystallographic studies of α-CA-s have shown that catalytic activity occurs in a cone-shaped hydrophobic pocket,^19^ where the active zinc ion, surrounded by three histidine groups, is located at the bottom of this pocket. However, the hydrophobic pocket is sufficiently large to accommodate compounds larger than CO_2_, as shown in Scheme 1b. Thus, the structural similarity between the carbonyl groups of CO_2_ and esters likely explains the high esterase activity of α-CA-s.^20–22^

**Scheme 1:**
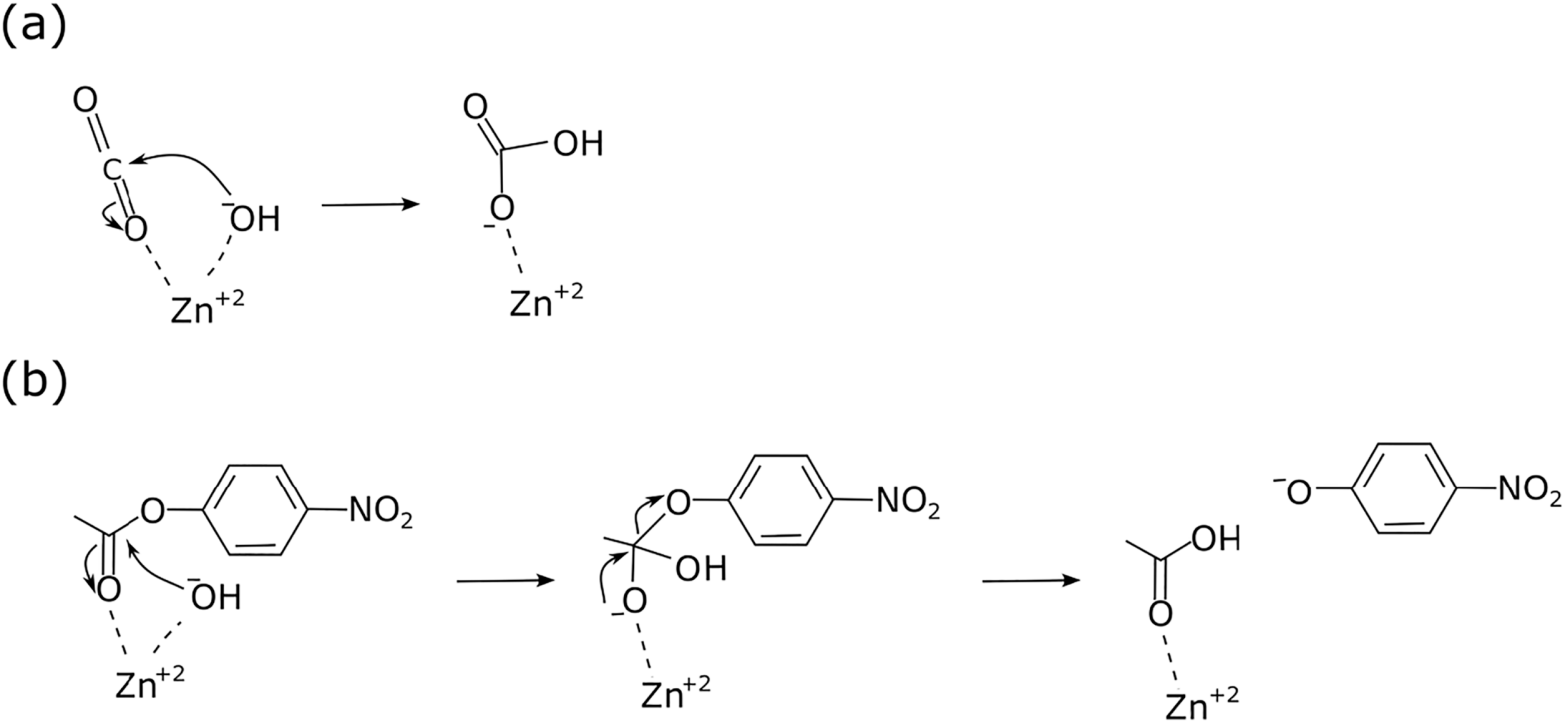
Catalytic Mechanisms of CA-s for (a) Hydration of CO_2_ (b) and Hydrolysis of the Ester *para*-Nitrophenyl Acetate (p-NPA)^22^ ^a^

a Adapted with permission from Ref 22. Copyright 2005 American Chemical Society.

Aminated mesoporous silica has been extensively studied for CO_2_ capture in gas–solid separation,^23–26^ and its synthesis can be fine-tuned so that the pores become large enough after amination to efficiently adsorb CA, which typically has a hydrodynamic diameter of 5 nm.^27–29^ Adsorption-induced stabilization of the enzyme is expected to occur. Mesostructured silicas with thin pore walls, such as MCM-41, typically exhibit poor hydrothermal stability.^30–32^ However, SBA-15 silica has thicker walls and a higher degree of polymerization within the pore walls, consequently increasing steam and thermal stability.^33,34^ It has been shown that increasing the hydrophobicity of silica upon functionalization with organic groups further stabilizes the adsorbents toward hydrothermal deterioration.^35,36^ Bui et al. suggested that the degree of functional groups, free silanol density and hydrophobicity play a significant role in the leaching process, and that a more hydrophilic surface leads to increasing leaching rates.^37^

Dispersions of SBA-15 functionalized with 3-(aminopropyl)-triethoxysilanes, denoted SBA-15-APTES, are suitable model systems for the thermal stabilization of CA, as the amino groups react with CO_2_, contributing to the capture capacity of the dispersions. The reaction chemistry of CO_2_-amine-water systems is well established for free alkanolamines, such as the industrial standard monoethanolamine (MEA), with amino group moieties analogous to SBA-15-APTES. While there are academic discussions concerning the detailed CO_2_–amine reaction mechanisms, it is clear that primary and secondary amines rapidly react with CO_2_ in water to form ammonium carbamate pairs as captured by Reaction 1.^38–40^ The ammonium carbamate pair can subsequently be hydrolyzed to form HCO_3_^–^, as shown in Reaction 2.^41^ Ammonium carbamate formation has a 2:1 stoichiometric amine-to-CO_2_ ratio, whereas HCO_3_^–^ formation has a 1:1 stoichiometric amine-to-CO_2_ ratio. The catalytic promotion of HCO_3_^–^ formation can thus increase the cyclic capacity and productivity of the scrubber fluid,^3,42–46^ however, without effective catalysis, the reaction rates of HCO_3_^–^ formation are often too slow to be relevant for industrial applications in CO_2_ capture.

1 $$2R-NH_{2}+CO_{2}⇌R-\mathrm{NHCO}O^{-}+R-NH_{3}^{+}$$

2 $$R-NH_{2}+CO_{2}+H_{2}O⇌R-NH_{3}^{+}+\mathrm{HC}O_{3}^{-}$$

It should be noted that analogous primary and secondary alkanolamines are used as catalytic or rate-promoting additives in scrubbing fluids, such as potassium carbonate;^42^ however, it is hypothesized that this autocatalytic feature would not be sufficiently effective in accelerating HCO_3_^–^ formation.

Building on the potential of adsorption-based thermal stabilization of CA in particle-based scrubbing fluids, we present a study on CA-s immobilized on amine-functionalized mesoporous silica for enzyme-accelerated HCO_3_^–^ formation. We studied the adsorption of two types of CA on SBA-15-APTES. Enzyme catalysis and thermal stabilization were studied and infrared (IR) spectroscopy was used to show that HCO_3_^–^ was formed with CA present.

## 2 Experimental Section

### 2.1 Carbonic Anhydrase

#### 2.1.1 Enzyme Adsorption

A stock solution of SBA-15-APTES particles was mixed with deionized water achieving a concentration of 1 mg-silica·mL^–1^. The suspension was sonicated for 10 min in a water bath sonicator to obtain a uniform suspension. Thermophilic CA from *Persephonella marina* (pmCA) and commercial bovine CA (bCA, Sigma-Aldrich) were prepared in deionized water at a concentration of 1 mg·mL^–1^. pmCA is found in hydrothermal vents in the deep-sea region of the Pacific Ocean and is naturally resilient to higher temperatures.^47^ Aliquots of the stock solutions of SBA-15-APTES and CA were mixed with deionized water, giving a total volume of 1 mL. The concentration of SBA-15-APTES was kept constant at 0.25 mg-silica·mL^–1^, while the final concentrations of pmCA or bCA varied between 10–400 μg·mL^–1^ (sorbent-enzyme mass ratios 1:0.4–16). The enzyme-SBA-15-APTES suspensions were incubated on a rotator at room temperature for 24 h to promote enzyme adsorption into the mesopores of SBA-15-APTES.

After incubation, the mixtures were centrifuged at 12,000*g* for 10 min, to obtain enzyme-loaded silica pellets. The supernatants, containing the free enzymes, were carefully removed to determine the free enzyme concentrations. The pellets were dried and used for the activity assays. The amount of enzyme adsorbed onto the silica was determined by measuring the protein concentration in the supernatants using the Bradford protein assay (Pierce Coomassie Protein Assay Kit, Thermo Fisher Scientific, Massachusetts, USA).^48^ A mass balance was performed on the split of the enzyme in the supernatant and the pellet to determine the adsorbed amount of enzyme on the silica. All samples were prepared in triplicate. Nonlinear regression analyses were conducted, and the resulting confidence intervals are included in the figures.

#### 2.1.2 Enzyme Activity Assay

The enzyme kinetics of pmCA and bCA were assessed using p-nitrophenyl acetate (p-NPA) as a substrate, following the method described by Armstrong et al.^49,50^ The reaction mixture was prepared in a 1 cm cuvette and consisted of 10 μL enzyme solution (10 μg·mL^–1^), 0.965 mL Tris-HCl buffer (50 mM, pH 7.6), and 0.25 mL of 3 mM p-NPA. Enzymatic activity was determined spectrophotometrically (GENESYS 150 UV–visible Spectrophotometer, ThermoFisher Scientific, USA) by measuring absorbance at 405 nm at 25 °C over a reaction time of 10 min. Michaelis–Menten kinetics were determined at room temperature and 65 °C using the following substrate concentrations: 1.0, 1.5, 2.0, 2.5, 3.0, 3.5, 4.0, 4.5, and 5.0 mM. To determine the optimum temperature for the kinetic activity of pmCA, the following temperatures were applied: 4, 25, 37, 45, 65, 80, and 90 °C.

#### 2.1.3 Enzyme Desorption Assay

CA with loadings of 70, 130, and 180 mg bCA·g-SBA-15-APTES^–1^ was immobilized as described above. After incubation, the mixture was centrifuged at 12,000*g* for 10 min to pellet the enzyme and substrate, and the supernatant was removed. The pellet was then mixed with deionized water by pipetting to form a uniform dispersion, which was kept in a refrigerator for 24 h. The enzyme concentration in the new supernatant was measured again, as described above.

#### 2.1.4 Enzyme Thermal Stability

Enzymes pmCA and bCA were adsorbed on SBA-15-APTES at two different loadings. These levels were 170 mg pmCA·g-SBA-15-APTES^–1^ and 260 mg pmCA·g-SBA-15-APTES^–1^ for pmCA, and 130 mg bCA·g-SBA-15-APTES^–1^ and 180 mg bCA·g-SBA-15-APTES^–1^ for bCA, corresponding to mass loadings of 6/9% and 13/19% of the maximum loadings. Relatively low loading was used to ensure that mass transfer limitations were not introduced by blocking the pores with excessively high enzyme loadings. The initial enzyme concentrations were 50 and 75 μg enzyme·mL^–1^. Immobilized enzymes were incubated at 40, 65, and 90 °C. For comparison, free pmCA and bCA were incubated under the same conditions as the adsorbed versions to study changes in thermal stability upon adsorption.

The residual CA activities were measured at different time intervals. Volumetric esterase activity was determined using the colorimetric p-NPA assay as a function of the hydrolysis rate of the substrate per unit time for CA adsorbed on aminated mesoporous silica.

#### 2.1.5 Speciation of Reaction Products

A concentrated dispersion of the SBA-15-APTES was prepared. 1250 mg of the stock dispersion, corresponding to 500 mg SBA-15-APTES, was added to Eppendorf tubes, to which 500 μL of 20 mg·mL^–1^ bCA or DI water was added to achieve the same final concentration. The samples were kept at 40 °C in a water bath with an insulating lid. The samples were then subjected to CO_2_ by blowing it over the liquid surface. The same mass flow of CO_2_ was ensured by splitting the inflow of gas so that the tests could be performed simultaneously. A droplet of each sample was collected at 1, 2, 5, 10, 15, 20, 25, and 30 min, and studied with a Varian 670-IR spectrometer equipped with an attenuated total reflection accessory immediately after the sample was taken. The CO_2_ loading was controlled by the CO_2_ contact time.

### 2.2 Mesoporous Silica Support

#### 2.2.1 Synthesis of SBA-15

SBA-15 was synthesized in an upscaled manner, based on previous literature.^27,51^ Initially, 40.0 g of the polymer Pluronic P123 [(EO)_20_(PO)_70_(EO)_20_, Sigma-Aldrich] was fully dissolved in 300 mL of water and 1200 mL of 2 M HCl [Sigma-Aldrich]. The solution was then transferred to a 2.5 L reactor equipped with a heating jacket (Hubber ministat 240), reflux condenser, and overhead stirrer (CAT R80D). The solution was heated to 40.0 °C under slow stirring for several hours. The synthesis of SBA-15 was initiated by adding 85.0 g of tetraethyl orthosilicate (TEOS, Merck, ≥99%), resulting in a TEOS:P123 molar ratio of 1:0.017. The solution was stirred vigorously for 10 min, followed by static aging for 24 h. This was followed by the thermal treatment of the formed gel, where the final reaction mixture temperature was 97 °C. These conditions were maintained for 24 h, after which the reaction mixture was cooled rapidly. The solids were filtered and washed until neutral pH was reached. The resulting silica was calcined overnight at 250 °C to ensure template removal.

#### 2.2.2 Amine Coating

The amine coating was performed according to a previously established method.^52,53^ The silica was degassed at 110 °C overnight to remove any preadsorbed moisture. Approximately 20 g of silica was then transferred to a three-necked bottle with a Dean–Stark reflux condenser containing 1200 mL of toluene [Sigma-Aldrich, 99.8%]. The reaction mixture was heated to 50 °C, and a catalytic amount of water was added. The mixture was maintained at 50 °C for an additional hour, after which the temperature was increased to reflux. The amine silane monomer (3-aminopropyl)­triethoxysilane (APTES, Sigma-Aldrich, ≥98%) was added in a 5:1 ratio to the estimated silanol group content of the silica. The reaction mixture was kept for 24 h, after which it was cooled and the solid was filtered off. The solid was washed with toluene and ethanol (VWR, ≥ 99.9%), and dried.

#### 2.2.3 Thermal Stability

The thermal stability of SBA-15-APTES was tested using two dispersions of 500 mg of SBA-15-APTES in deionized water. One sample was kept as is, whereas the other was loaded with CO_2_. The autoclaves were carefully closed and maintained at 90 °C for 14 d. The samples were subsequently cooled and dried. X-ray diffraction (XRD) was performed on the samples to determine the presence of partial or full collapse of the pore structure. Leaching of the amines from the SBA-15-APTES when suspended in water was tested using ^1^H and ^13^C NMR. SBA-15-APTES was suspended in D_2_O and incubated at 90 °C for 2 weeks, both as lean and CO_2_-loaded preparations.

### 2.3 Material Characterization

A Panalytical X’Pert Pro diffractometer (Cu–Kα radiation) in transmission mode operated at 40 mA and 45 kV was used to collect XRD patterns of the SBA-15 and the SBA-15-APTES samples in the range 0.5 < 2θ < 5°. Scanning Electron Microscopy (SEM) images were collected using a JEOL JSM-7000F microscope operating at a 10 mm working distance and 2 kV with SBA-15 spread on Oxford aluminum stubs covered with carbon tape. Transmission Electron Microscopy (TEM) images were taken using a JEOL- JEM-2100 microscope with a Schottky-type field emission gun operating at 200 kV accelerating voltage and a Gatan Ultrascan camera. 1–2 μg samples were dispersed in 2–3 mL of ethanol using a sonicator for 2–3 min. A drop of the suspension was added to a Cu grid and left to dry at room temperature. CO_2_ and N_2_ adsorption isotherms were measured using a Micrometrics ASAP 2020 adsorption analyzer at 20 °C (CO_2_ > 99.9995%, Linde Gas Company­(AGA)) and – 196 °C (N_2_ > 99.999%, Strandmöllen) respectively. SBA-15-APTES samples were degassed under high dynamic vacuum at 130 °C for 10 h, while regular SBA-15 silica was degassed at 200 °C. The Brunauer–Emmett–Teller (BET) surface area was determined in the relative pressure range of p/p^0^ = 0.05–0.20, and the pore volume was determined at *p*/*p* ^0^ = 0.99. The pore size distribution was determined using density functional theory (DFT). Thermogravimetric analysis was performed on a TA Instruments Discovery analyzer. The samples were heated from room temperature to 800 °C in a platinum cup, in a N_2_ atmosphere, at a rate of 15 °C/min. A Zetasizer Nano ZS was used to measure the zeta potential and hydrodynamic radius. A dip cell was used to determine the zeta potential. ^1^H and ^13^C NMR spectra were recorded using a Bruker Avance 400 MHz spectrometer.

## 3 Results and Discussion

### 3.1 Free Enzyme Esterase Activity

We hypothesized that the pmCA would exhibit high esterase activity at elevated temperatures. The volumetric enzyme activity of free pmCA was determined at room temperature and 65 °C (Figure 1a,b). The esterase activity followed Michaelis–Menten behavior at room temperature, reaching maximum volumetric activity at a substrate concentration of 3 mM p-NPA. This maximum was well within the range of 1–5 mM reported in previous kinetic studies of carbonic anhydrase.^54^ The rate-determining step of the reaction is the binding of p-NPA to pmCA, forming a pmCA–p-NPA complex. However, the volumetric esterase activity recorded at 65 °C did not exhibit the typical Michaelis–Menten behavior (Figure 1b). Enzyme-binding kinetics can also occur via, for example, multisubstrate reactions, including random, ordered, and ping-pong mechanisms.^55^ In this context, it can be noted that certain versions of CA have been shown to exhibit non-Michaelis–Menten kinetics in different buffers. Previous literature suggested that the non-Michaelis–Menten behavior arose because of the formation of a stable enzyme-HCO_3_^–^ complex, which could have decomposed in a manner that depended on the proton availability of the buffer^20^ or that the HCO_3_^–^ interacted with at least two different forms of CA with different ionizing groups in the vicinity of the active site.^56^ Enzymes can undergo abrupt changes in kinetic parameters at increasing temperatures due to disrupted conformational landscapes, resulting in kinetic regimes inconsistent with Michaelis–Menten behavior.^57–61^ However, we cannot confirm whether this observed behavior arises from such mechanisms or from large deviations in experimental data without rigorous experimental data.

**Figure 1:**
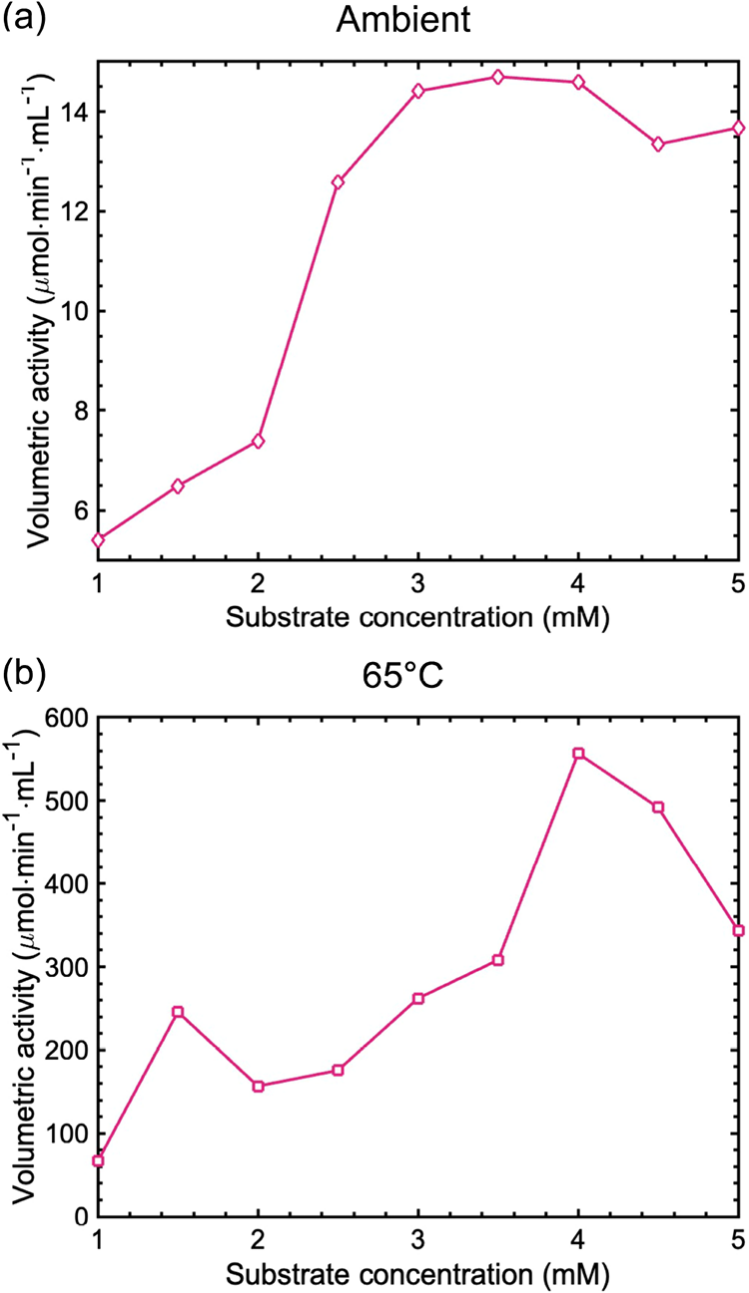
Michaelis–Menten curves of free pmCA using substrate p-NPA at (a) room temperature and (b) 65 °C. Lines between data points are used as a guide for the eye.

The temperature-dependent volumetric esterase activity for free pmCA increased with temperature, which is normal for enzymes (Figure 2). However, a local minimum in esterase activity was observed at 45 °C, after which it increased drastically. Maximum activity was observed at 65 °C, and decayed at higher temperatures. The local minimum observed in the activity–temperature relationship could be an effect of non-Michaelis–Menten behavior. After establishing the enhanced stability of pmCA, we evaluated the physical characteristics of the SBA-15-APTES support to understand its contribution to enzyme stabilization.

**Figure 2:**
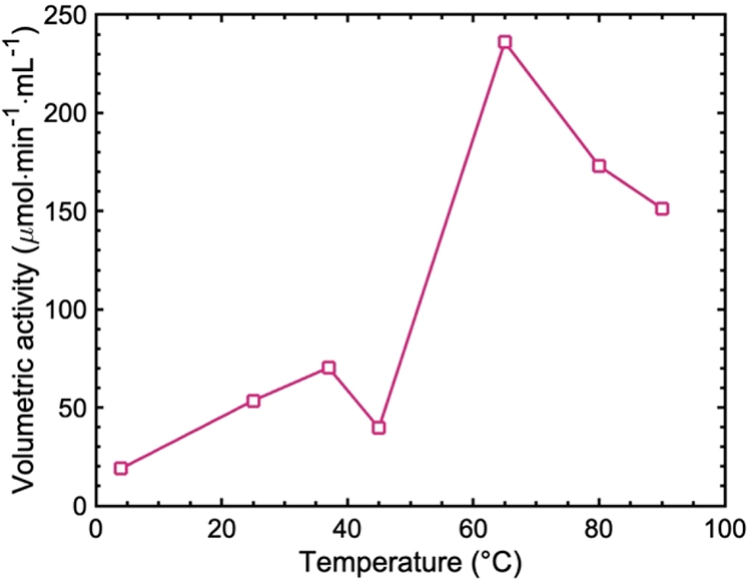
Enzyme esterase activity of free pmCA at various temperatures with a p-NPA concentration of 3 mM. Lines between data points are used as visual aids.

### 3.2 Materials Properties of the Aminated SBA-15-APTES

It was hypothesized that SBA-15-APTES would have pores larger than the effective hydrodynamic dimension of CA. CA adsorption onto SBA-15-APTES is illustrated in Figure 3a. The BET surface area, pore size, and pore volume of the as-synthesized SBA-15 and SBA-15-APTES were determined using N_2_ adsorption data (Figure S1a–b). For the as-synthesized SBA-15 silica, the surface area and pore size were 1060 m^2^·g^–1^ and 11.8 nm, respectively, and the pore volume was 1.3 cm^3^·g^–1^. For SBA-15-APTES, the corresponding BET surface area was 492 m^2^·g^–1^, pore size was 9.6 nm, and pore volume was 0.8 cm^3^·g^–1^.

**Figure 3:**
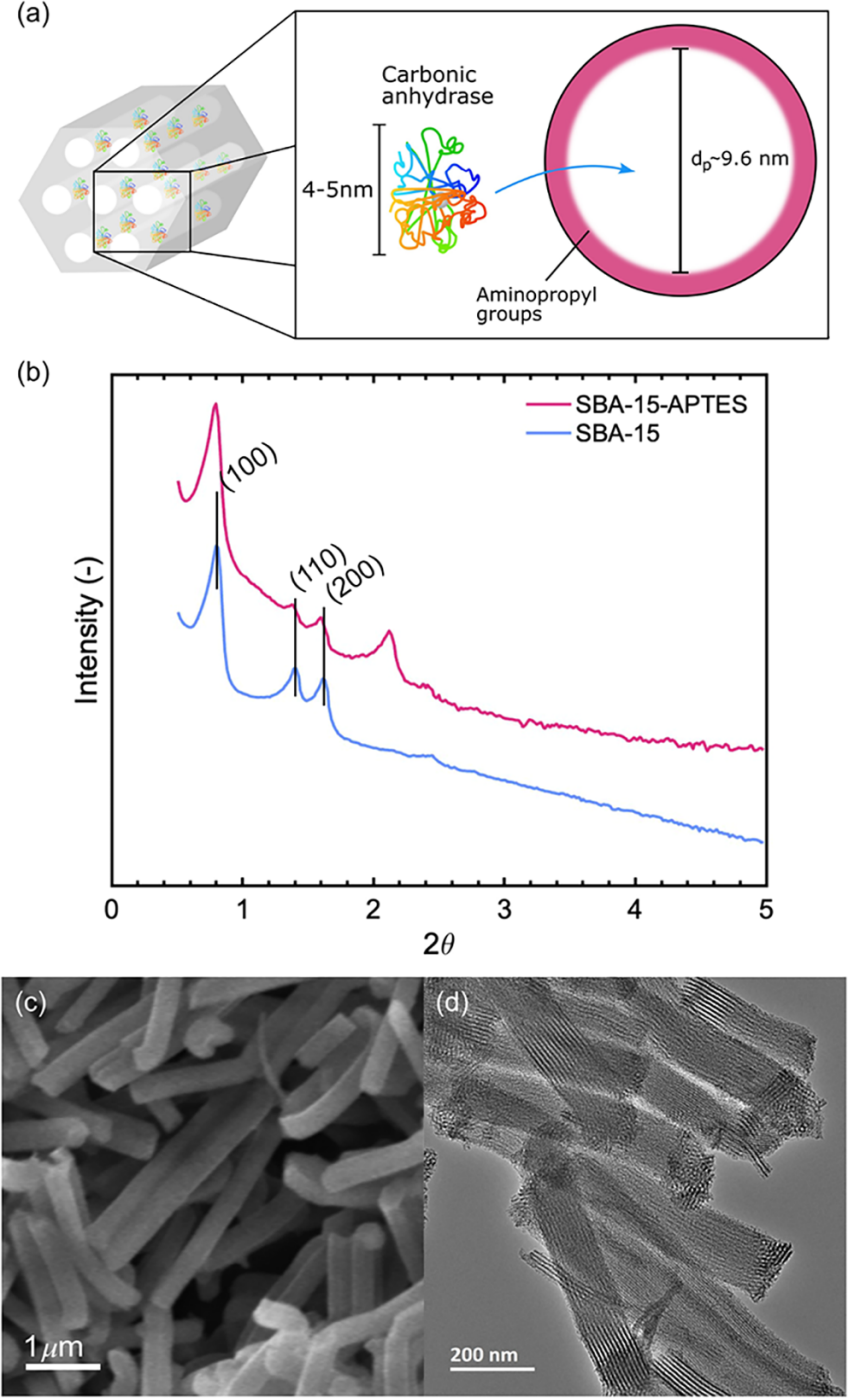
(a) Illustration of the adsorption of carbonic anhydrase onto aminated SBA-15-APTES, (b) X-ray diffraction pattern of calcined SBA-15 and SBA-15-APTES, (c) scanning electron microscopy images of calcined SBA-15 particles demonstrating their rod-like morphology, and (d) transmission electron microscopy images of SBA-15 particles, with tubular pores running along the particle length.

The CO_2_ adsorption on SBA-15-APTES (Figure S2a–b) revealed a typical chemisorption behavior with a plateau in the low-pressure region. The functionalization of SBA-15 to SBA-15-APTES was further confirmed by thermogravimetric traces (Figure S2c), from which an amino group density of 2.5 NH_2_·nm^–2^ was determined. Further details concerning the characterization of SBA-15 and SBA-15-APTES are provided in the Supporting Information.

The surface area, pore size, and pore volume decreased after amination because of the introduction of organic groups into the pore structure. To confirm that the enzymes could physically enter the pores, their hydrodynamic diameters were determined at a concentration of 1 mg·mL^–1^. pmCA had a hydrodynamic diameter of 5.9 ± 1.2 nm while bCA had a hydrodynamic diameter of 4.2 ± 0.2 nm, see Table 1, in accordance with previous literature values in the range of 5 nm.^28,29^

**Table 1:** Hydrodynamic Diameters of Thermophilic Carbonic Anhydrase (pmCA) and Commercial Bovine Carbonic Anhydrase (bCA)

	hydrodynamic diameter (nm)
pmCA	5.9 ± 1.2
bCA	4.2 ± 0.2

The *p6 mm* hexagonal structure of the calcined SBA-15 was confirmed by XRD, with three distinct reflections, 100, 110, and 200 (Figure 3b).^27^ Differential peak intensities for SBA-15 and SBA-15-APTES were assigned to X-ray contrast differences, and it was concluded that the crystallographic order remained intact after the amination. SEM and TEM images (Figure 3c,d) were used to examine the particle morphologies and pore structures. The particles clearly showed a rod-like shape (Figure 3c), with tubular mesopores running along their length (Figure 3d), offering suitable space for the enzyme to occupy.

### 3.3 Enzyme Adsorption and Capacity

As CA was established to be sufficiently small to adsorb effectively within SBA-15-APTES, the adsorption isotherms of pmCA and bCA on dispersed SBA-15-APTES were determined. From the adsorption isotherms (Figure 4a,b), maximum adsorption capacities of 1 400 ± 200 mg pmCA·g-SBA-15-APTES^–1^ and 2 100 ± 500 mg bCA·g-SBA-15-APTES^–1^, and Langmuir constants of 11.4 ± 3.9 dm^3^·mol^–1^ and 3.5 ± 1.2 dm^3^·mol^–1^ were derived for pmCA and bCA respectively. Considering the large specific mass of CA adsorbed on SBA-15-APTES, both pmCA and bCA adsorbed within the pores. The higher Langmuir constant for pmCA than that for bCA indicates a higher affinity for SBA-15-APTES. This difference is consistent with the larger hydrodynamic radius for pmCA, as shown in Table 1. Care is advised in the analysis; notably, the Langmuir model only partially describes the adsorption behavior of bCA and pmCA. The comparably large parameter errors and small error bars in the data at low concentrations suggest deviation from the single-component Langmuir adsorption model. The desorption of bCA was tested after immersing SBA-15-APTES, with preadsorbed bCA, in deionized water for 24 h. After separating the pellet and supernatant, no bCA was detected in the supernatant, indicating irreversible CA adsorption.

**Figure 4:**
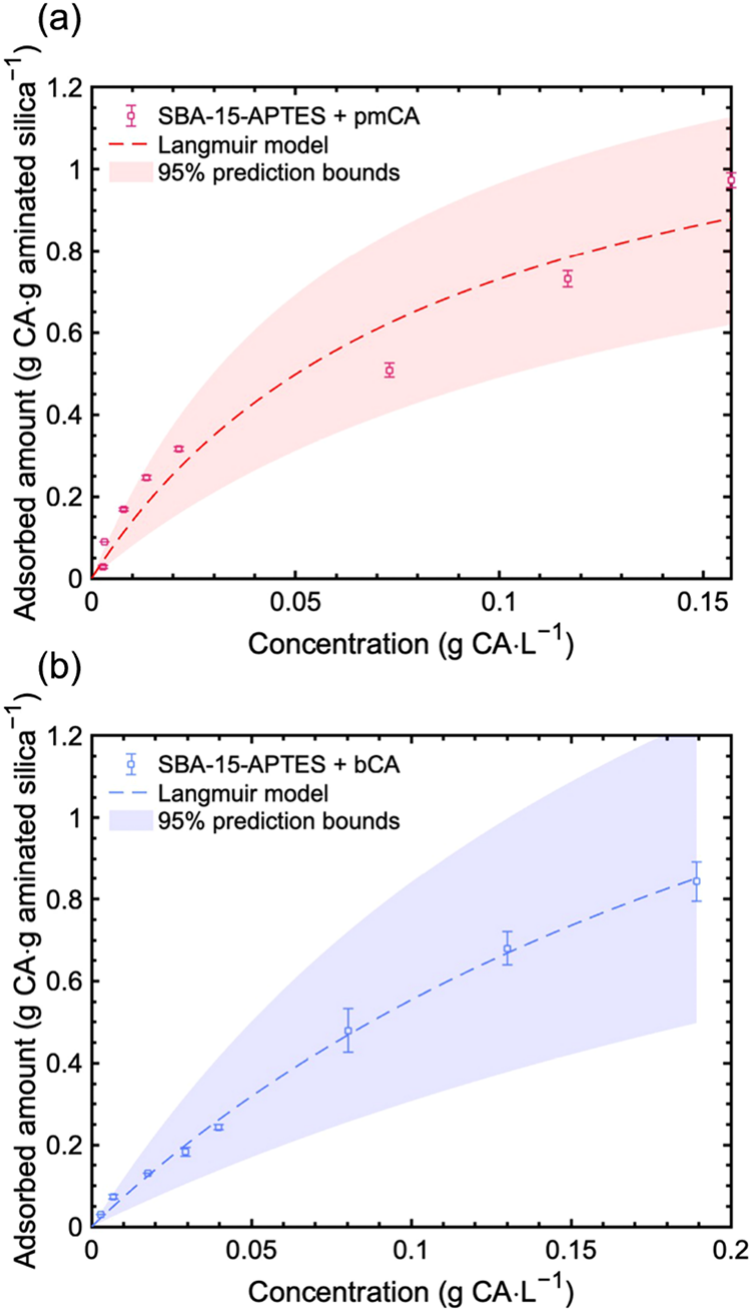
Enzyme adsorption isotherms: (a) thermophilic (pmCA) and (b) commercial bovine (bCA) carbonic anhydrase on SBA-15-APTES at room temperature. Nonlinear regression using the Langmuir adsorption isotherm model was used, and the 95% confidence interval is displayed.

### 3.4 Enzyme Assays and Thermal Stability of Adsorbed Enzymes

The hypothesis in this context is that CA is thermally stabilized by adsorption on SBA-15-APTES. As shown in Figure 5, the residual esterase activities of the adsorbed pmCA and bCA were effectively retained at 40 and 65 °C for 14 d. At 90 °C, both pmCA and bCA retained their activities for 4 d. The half-life activities of both enzymes immobilized on SBA-15-APTES were approximately 7 d at 90 °C. Residual activity is defined as the ratio of the volumetric activity at time *t* to the initial activity at *t* = 0. Because p-NPA hydrolysis occurs at the same active site as CO_2_ hydration, enzymatic CO_2_ activity can be inferred from esterase activity.^62^

**Figure 5:**
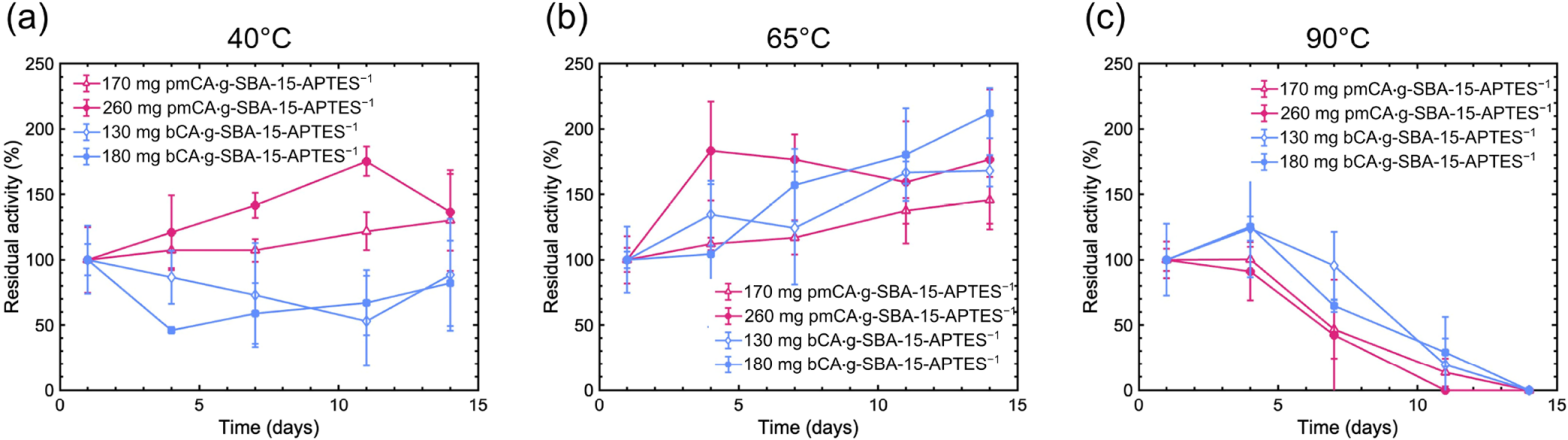
Relative residual enzymatic activity of thermophilic (pmCA) commercial carbonic (bCA) anhydrase immobilized on SBA-15-APTES with starting enzyme concentration 50 μg·mL^–1^ and 75 μg·mL^–1^ incubated at (a) 40 °C, (b) 65 °C, and (c) 90 °C for 14 d.

For comparison, free pmCA and bCA were incubated at the same temperatures. Here, a complete loss of activity was observed within the first 4 d at all temperatures studied, and the activity data are presented in Figure S3a–c for 40–90 °C. This aligns with previous studies that reported an almost complete loss of residual activity of erythrocyte bCA after just 40 min at 65 °C.^63^

Low CA loading was selected to prevent pore blockage. Since diffusion within the pore network depends on both pore size and tortuosity, the introduction of enzymes can naturally restrict transport.^64,65^ Care must therefore be taken to avoid creating diffusion limitations, as this would render part of the enzyme population inactive when substrates are consumed before reaching the inside of the matrix.

Adsorbed pmCA and bCA retained high esterase activities at 40 and 65 °C for the study period, demonstrating enhanced thermal stabilities compared to free CA. Notably, bCA that normally denatures and deactivates at these temperatures displayed an activity similar to that of the thermophilic pmCA. Structural reorganization of CA upon adsorption could explain the observed behavior. The initial activities are presented in Table 2. As expected,^66^ higher temperatures resulted in an increased initial activity.

**Table 2:** Initial Volumetric Activities of pmCA and bCA Immobilized on SBA-15-APTES Incubated at Different Temperatures

adsorbed amount CA on SBA-15-APTES	40 °C (μmol·mL^–1^·min^–1^)	65 °C (μmol·mL^–1^·min^–1^)	90 °C (μmol·mL^–1^·min^–1^)
170 mg pmCA·g-SBA-15-APTES^–1,^ ^a^	280 ± 10	450 ± 40	1 030 ± 90
260 mg pmCA·g-SBA-15-APTES^–1,^ ^b^	230 ± 60	310 ± 60	980 ± 140
130 mg bCA·g-SBA-15-APTES^–1,^ ^a^	230 ± 30	320 ± 20	1 050 ± 20
180 mg bCA·g-SBA-15-APTES^–1,^ ^b^	250 ± 70	330 ± 80	1 100 ± 300

a Initial concentration: 50 μg CA·mL^–1^

b Initial concentration: 75 μg CA·mL^–1^.

Interactions between a surface and an enzyme have been shown to impose conformational changes in the enzyme structure. The nature of the interaction is typically weak, and noncovalent with a free energy of adsorption of 1.5–3 kJ/mol with relatively few contact points.^67–70^ Yet, several studies have shown that the enzyme flexibility remains low in the adsorbed state at elevated temperatures, up to 133 °C, largely explaining the increased thermal stability achieved by adsorption.^71–73^

The binding of the hydrophobic dye 8-anilino-l-naphthalenesulfonic acid (ANS) to free and adsorbed pmCA and bCA was studied to assess conformational changes in CA upon adsorption. Adsorbed pmCA and bCA showed a red shift and decreased fluorescence intensity compared to the free enzyme (Figure S6 and Table S2). This indicates that CA did not denature on adsorption. Denaturation would have increased the contact area between the hydrophobic chains and the solvent, consequently, the added hydrophobic ANS dye would have had an increased interaction, leading to a blue shift and increased intensity of the emission spectrum.^74^

### 3.5 Thermal Stability of SBA-15-APTES

It is known from the literature that the stability of aminated mesoporous silica^30–32^ is somewhat limited. In light of this and the reduced activity of the adsorbed pmCA and bCA at 90 °C, we studied the structural integrity of SBA-15-APTES under hydrothermal treatment. XRD data were recorded for fresh SBA-15-APTES and samples that had been hydrothermally treated in dispersion at 90 °C for 14 d, in lean- and CO_2_-loaded states. The XRD data are shown in Figure 6. The defined XRD reflections for SBA-15-APTES disappeared after the extensive hydrothermal treatment. The presence of CO_2_ did not alter the degradation. Amine leaching of APTES, the structure of which is presented in Figure S4a, was studied using analyses of ^13^C (Figure S4b) and ^1^H (Figure S4c) NMR spectra recorded in the liquid phase.

**Figure 6:**
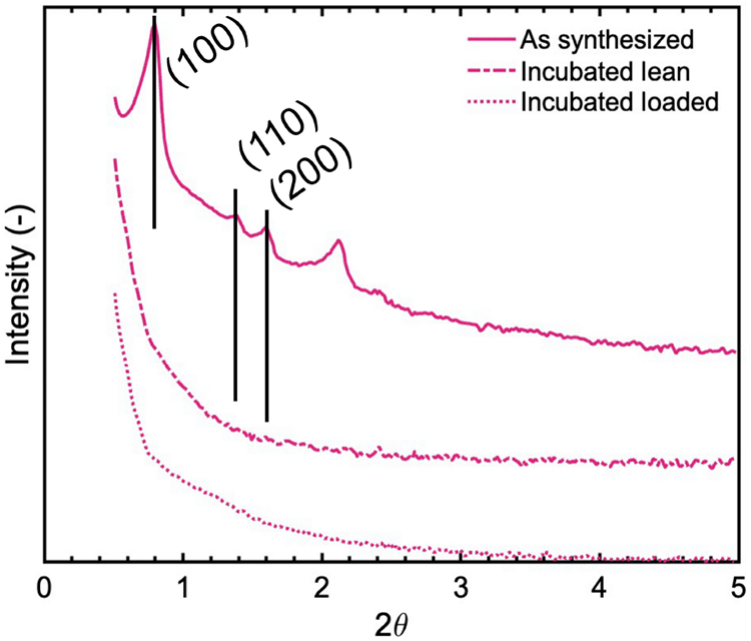
X-ray diffraction data for SBA-15-APTES as-synthesized, and incubated in dispersion at 90 °C for 14 d, lean and loaded with CO_2_.

The hydrothermal stability of mesoporous silica is linked to the degree of condensation and the thickness of the pore walls. Protection against siloxane hydrolysis can be achieved by reducing the silanol content. Raising the temperature of the hydrothermal treatment during the synthesis can significantly improve the hydrothermal stability but may necessitate the use of thermally stable fluorocarbon polymers. Alternatively, metal ions can be incorporated into the matrix to increase the thermal stability. Li et al. demonstrated a highly stable mesoporous silica with aluminum atoms incorporated within the pore wall matrix. After 300 h of boiling, the aluminosilica material exhibited an XRD pattern that was consistent with an intact structure.^75–77^ Alternatively, silanol capping by trimethyl groups been shown to enhance the thermal stability of mesoporous silica.^78^

### 3.6 Speciation of HCO_3_^–^ and Ammonium Carbamate Ion Pars in Aqueous Dispersions

It is expected that adsorbed CA would enhance the rate of HCO_3_^–^ formation on dispersed SBA-15-APTES particles. It should be noted that CA is a catalyst for CO_2_ hydration, and only affects the rate of HCO_3_^–^ formation, but not the equilibrium concentrations of carbamate and HCO_3_^–^. However, the hydrolysis of carbamates yielding HCO_3_^–^ has a high activation energy (150 kJ/mol for MEA) and proceeds at rates too slow to be of industrial relevance.^79–81^ Strictly speaking, CA does not influence the available fractions of carbamates and HCO_3_^–^ if the absorption time is long enough to reach equilibrium. However, from an industrially applied perspective, it is shown here that the adsorbed CA increased the available HCO_3_^–^ fraction at the time scale of an industrial absorption process.

The speciation of the ammonium carbamate moieties and HCO_3_^–^ in the reacting dispersions with CO_2_ was studied using IR spectroscopy, and the resulting spectra are presented in Figure 7. The bands at 1493/1552 cm^–1^ were assigned to the ammonium carbamate moieties, while the bands at 1357/1614 cm^–1^ were assigned to HCO_3_^–^.^82–84^ Based on this assignment, it was evident that ammonium carbamate moieties formed on SBA-15-APTES without CA and HCO_3_^–^ was not detected. For SBA-15-APTES with CA, additional HCO_3_^–^ peaks were detected at 1357 and 1614 cm^–1^. HCO_3_^–^ formation was particularly prominent at high CO_2_ loadings. Although the measurements were not quantitative in nature, it can be observed in Figure 7b that the relative band intensities for HCO_3_^–^ to ammonium carbamates increased with time and CO_2_ loading. This showed thatHCO_3_^–^ formation was prominent in the CA-catalyzed system, in contrast to the noncatalyzed system.

**Figure 7:**
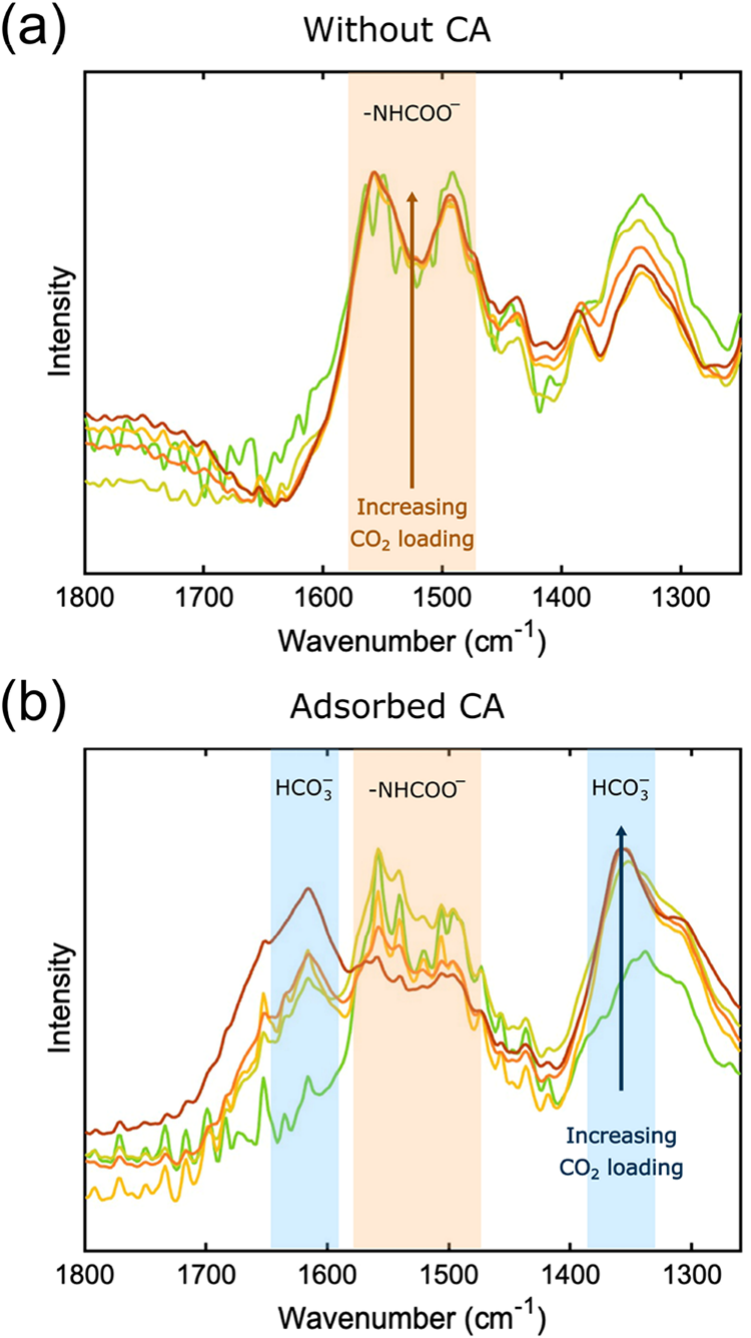
Infrared spectra of SBA-15-APTES dispersions during reactions with CO_2_ (a) without carbonic anhydrase (CA) and (b) catalyzed by adsorbed CA. Coloring from green to red denotes increased CO_2_ loading.

The viscosity of the dispersion decreased upon adsorption of CA, which likely further increased the formation of ammonium carbamate moieties on SBA-15-APTES by increasing the diffusion rate of CO_2_ throughout the dispersion. It can be noted that the rates of formation of ammonium carbamate moieties and HCO_3_^–^ will not only depend on the presence and absence of CA but also on the partial pressure of CO_2_ near the dispersion surface, temperature, and amine density and availability. While CO_2_ loading is controlled by the contact time, the mass flow rate of the CO_2_ gas also influences the extent of loading in the dispersion. A low flow rate and split stream of CO_2_ were used for contact with the samples and to ensure comparable driving forces. Conversely, at higher gas flow rates, the reaction products observed at the same time intervals differed. Therefore, the time scale and observed product distribution should not be directly converted into absolute reaction rates. Instead, they served as observables to probe the differences between samples that were exposed to the same conditions. In this case, the flow of CO_2_ was deliberately kept small to allow the observation of the formed HCO_3_^–^ catalyzed by the CA in one of the samples. At early contact times, ammonium carbamate moieties and HCO_3_^–^ were not detected, and the spectra are presented in Figure S5.

## 4 Conclusion

Bovine (bCA) and thermophilic (*P. marina*, pmCA) carbonic anhydrases were adsorbed on dispersed SBA-15-APTES particles and showed high activity for over 14 d at 40 and 65 °C, and for 4 d at 90 °C. Free enzymes displayed a reduced activity within the first 4 d at the same temperature, demonstrating the increased thermal stability achieved by the adsorption. Free pmCA showed defined non-Michaelis–Menten kinetics at 65 °C. IR spectroscopy was used to show that HCO_3_^–^ and ammonium carbamate ion pairs were formed in aqueous dispersions of SBA-15-APTES with adsorbed CA. In the absence of CA, only ammonium carbamate ion pairs were detected. SBA-15-APTES demonstrated high enzyme loading capacities, reaching maximum values of 1400 ± 200 mg pmCA·g-SBA-15-APTES^–1^ and 2100 ± 500 mg bCA·g-SBA-15-APTES^–1^. For our experiments, however, lower loadings were deliberately chosen to avoid diffusion limitations typically associated with high pore filling. These experimental loadings were 170 and 260 mg pmCA·g-SBA-15-APTES^–1^ (corresponding to 6% and 9% of the maximum pmCA loading) and 130 and 180 mg bCA·g-SBA-15-APTES^–1^ (13% and 19% of the maximum bCA loading), respectively. The physisorption of CA was irreversible, suggesting a strong interaction between the enzymes and SBA-15-APTES. The SBA-15-APTES lost its regular *p*6*mm* hexagonal mesoporous structure after hydrothermal treatment at 90 °C for 14 d.

The key result of this study is that bCA and pmCA were thermally stabilized by physical adsorption on SBA-15-APTES, and the temperature range for CA activity was significantly extended. Free CA typically deactivates rapidly at 65 °C.^63^ This supports our hypotheses that CA adsorption on internal pore surfaces improves thermal enzyme stability, and that adsorbed CA accelerates the formation of HCO_3_^–^. We could not establish the exact extent of the stability enhancement because of the limited hydrolytic stability of SBA-15-APTES.

For future studies, we envision investigating why CA does not exhibit traditional Michaelis–Menten kinetics at all temperatures, as well as studies of mutated and adsorbed CA. Additional studies on the use of adsorbed CA in combination with scrubbing liquids for BECCS are envisioned, but colloids with higher hydrothermal stability are required.

## Supplementary Material



## Notes

S1: Characterization of SBA-15 and SBA-15-APTES. N_2_ sorption isotherm, DFT pore size distribution, CO_2_ adsorption at 20 °C, TG analysis. S2: Free enzyme stability. S3: Amine leaching test ^13^C and ^1^H NMR spectra. S4: Additional infrared (IR) spectra of aminated dispersions contacted with CO_2_. S5: Fluorescence spectra of adsorption of hydrophobic dye (ANS) (PDF)

## Abbreviations

APTES: 3-(aminopropyl)­triethoxysilane
BET: Brunauer–Emmett–Teller
bCA: bovine erythrocyte carbonic anhydrase
CA: carbonic anhydrase
DFT: density functional theory
p-NPA: paranitrophenyl acetate
pmCA: *Persephonella marina* carbonic anhydrase
XRD: X-ray diffraction
