# Supplementary material for: Accelerated Uptake of CO2 Catalyzed by Immobilized Thermophilic Carbonic Anhydrase on Dispersed Aminated Mesoporous Silica
Source: ACS Appl Mater Interfaces. 2025 Oct 28;17(45):61919–28. doi: 10.1021/acsami.5c08889 (PMC12616606; doi:10.1021/acsami.5c08889)
Supplement: Supplementary file 1 [file am5c08889_si_001.pdf]

# Supporting information

# Accelerated uptake of $CO_2$ catalyzed by immobilized thermophilic carbonic anhydrase on dispersed aminated mesoporous silica

Maja-Stina Svanberg Frisinger[1,‡], Didem Mimiroglu[1, 2, ‡], Latif Ullah[1], Swati Verma[3], Mats Martinelle[3], Per Berglund[3], Niklas Hedin[1,*]

1: Department of Chemistry, Stockholm University, SE-10691 Stockholm, Sweden

2: Department of Biochemistry, Faculty of Science, Sivas Cumhuriyet University, 58140, Sivas, Turkey

3: Department of Industrial Biotechnology, KTH Royal Institute of Technology, AlbaNova University Center, SE-106 91 Stockholm, Sweden

‡: Authors contributed equally

Corresponding author: *, niklas.hedin@su.se

## S1 Characterization aminated SBA-15-APTES

Surface area, pore size and pore volume of the calcined SBA-15 silica were investigated using $N_2$ adsorption/desorption analysis with the resulting isotherm presented in Figure S1a-b. A typical type IV isotherm with an H1 hysteresis loop is seen in Figure S1a, resulting from the mesoporous structure of SBA-15. The BET surface area was 1060 $m^2 \cdot g^{-1}$, and DFT-based average pore size was 11.8 nm, and total pore volume was 1.3 $cm^3 \cdot g^{-1}$. After functionalization the surface area was reduced to 492 $m^2 \cdot g^{-1}$, and the pore size was 9.6 nm and corresponding pore volume of 0.8 $cm^3 \cdot g^{-1}$, data not shown. Pore size and volume after functionalization remained high, and were larger than traditional non-functionalized SBA-15, which is typically reported in the range of 6-9 nm.[3,4] This ensures that the pores could accommodate the carbonic anhydrase after amine coating.

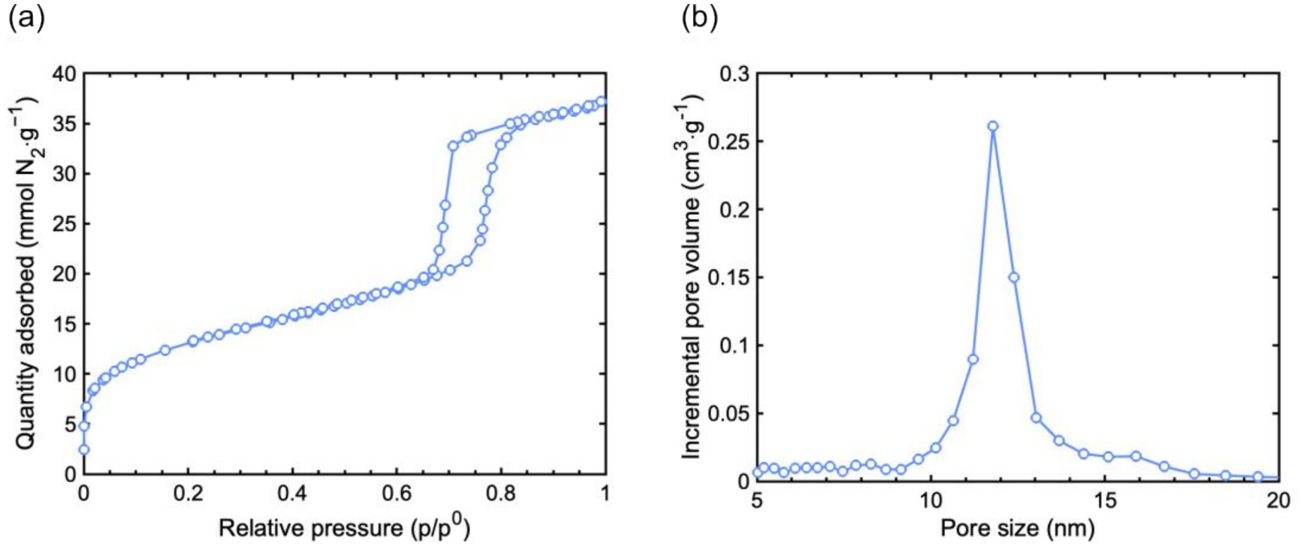


Figure S1. The results of the analysis of the $N_2$ sorption data at −196°C (a) the $N_2$ adsorption and desorption isotherms on the SBA-15 sample, showing a typical type IV adsorption isotherm with an H1 hysteresis loop and (b) the DFT pore size distribution for the calcined SBA-15 samples, centered around a pore size of 11.8 nm.

*Aminated SBA-15-APTES particles*

An adsorption isotherm of the SBA-15-APTES was collected as a measure of the $CO_2$ affinity and the success of the amine grafting, with resulting data presented in Figure S2a-b in a linear and semilogarithmic manner. The SBA-15-APTES shows behavior typical of chemisorption reaching almost its fullest capacity at relatively low pressure, (Figure S2a). This is a behavior investigated in our previous studies on $CO_2$ adsorption on aminated SBA-15 and other type of silica.[5–8] A semilogarithmic plot of the adsorption isotherm in the low-pressure region is presented in Figure S2b to enhance the resolution of the adsorption in this region. Amine group content on the SBA-15-APTES was investigated using thermogravimetric analysis (TG), with the resulting graph presented in Figure S2c. Weight loss in the region of 200–800°C corresponds to the thermal decomposition of the organic groups on the silica, which was used to estimate the amine group content. An amino group content of 2.5 $NH_2 \cdot nm^{-2}$ was determined. Pure SBA-15 silica displayed a steep slope in the range of 50–100°C corresponding to pre-adsorbed moisture. One should note that the samples were degassed at 110°C before being subjected to thermal treatment in the TG but still showed a high moisture content, owing to the highly hygroscopic nature of the pure SBA-15. The observed weight decrease of SBA-15 in the range 150-800°C is ascribed to the condensation of silanol

groups, forming siloxane bonds. The range of organic decomposition overlaps with that of silanol group condensation, but cannot be separated. This resulted in a slight overestimation of the amino group density of the SBA-15 APTES sample.

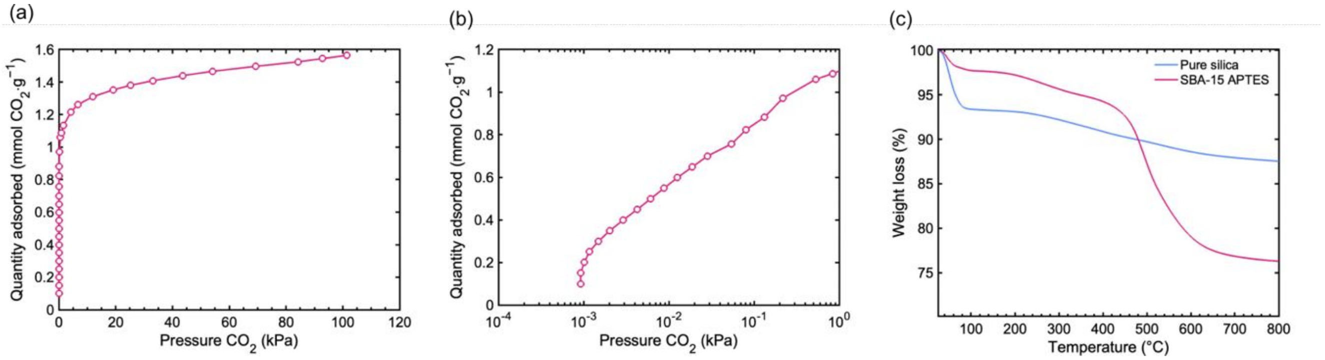


Figure S2. The $CO_2$ adsorption isotherm on the aminated samples SBA-15 APTES at 20˚C (a) linear isotherm (b) a semilog isotherm in the low-pressure region and (c) the resulting curve of thermogravimetric analysis of the aminated sample SBA-15-APTES and the pure silica sample SBA-15.

## S2 Free enzyme stability

Free pmCA and bCA were incubated to study the enhanced thermal stability compared to the adsorbed enzyme. The resulting data are presented in Figure S3. As can be seen, there was a loss of activity observed within the first four days. The activity at *t=0* is presented in Table S1.

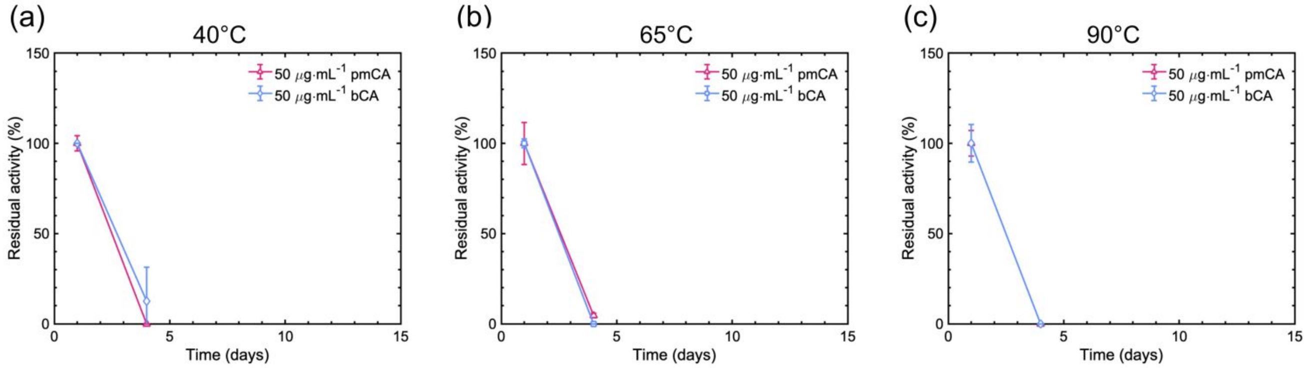


Figure S3. The residual relative enzymatic activity of free thermophilic (pmCA) commercial carbonic (bCA) anhydrase at enzyme concentration 50 $\mu$g·mL$^{-1}$ incubated at (a) 40˚C, (b) 65˚C, and (c) 90˚C for 4 days.

Table S1. The initial volumetric esterase activities of free thermophilic (pmCA) commercial carbonic (bCA) anhydrase at 50 $\mu$g·mL$^{-1}$ at 40˚C, 65˚C, and 90˚C.

| | pmCA | bCA |
|---|---|---|
| 40˚C ($\mu$mol·mL$^{-1}$·min$^{-1}$) | 24 ± 1 | 16 ± 2 |
| 65˚C ($\mu$mol·mL$^{-1}$·min$^{-1}$) | 86 ± 10 | 240 ± 3 |

| | | |
|---|---|---|
| 90˚C ($\mu$mol·mL$^{-1}$·min$^{-1}$) | 14 ± 10 | 58 ± 6 |

**S3 Amine leaching**

Leaching of the amines from the SBA-15-APTES when suspended in water was tested using liquid state $^1$H and $^{13}$C NMR spectroscopy. SBA-15-APTES was suspended in $D_2O$ and incubated at 90˚C for two weeks both as lean and $CO_2$-loaded dispersions. A schematic representation of hydrolyzed APTES, the expected leaching product, is presented in Figure S4a. The resulting NMR spectra are presented in Figure S4b-c below. Peaks in $^{13}$C NMR spectra are assigned to the carbons in the propyl chain, indicating that leaching of the amine was possible. However, unidentified peaks in the $^1$H NMR spectrum, Figure S4c indicate that other unknown compounds were present as well. Thermal decomposition of analogous MEA likely proceeds via carbamate polymerization, resulting in a complex mixture of products, we infer that a similarly complex mixtures may occur here.[9]

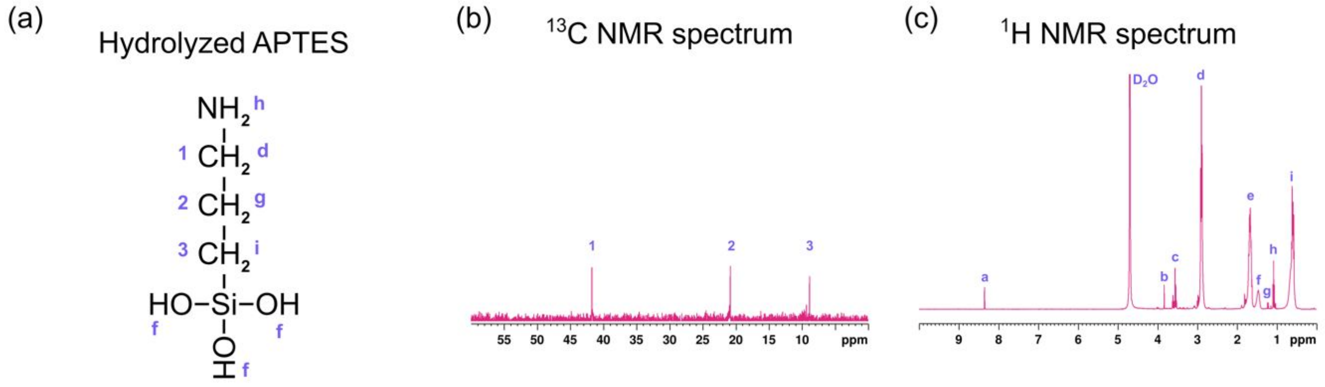


Figure S4. (a) chemical structure of hydrolyzed APTES (b) $^{13}$C NMR spectrum and (c) $^1$H NMR spectrum of supernatant of SBA-15-APTES in $D_2O$.

**S4 IR-spectra**

Supplementary IR spectra of the aminated dispersion with and without CA at early $CO_2$ contacting times are presented in Figure S5. The band at 1650 cm$^{-1}$ observed in Figure 2a and 2b was assigned to water, arising due to inhomogeneities in sample concentration. Owing to the high water content in the dispersion without CA, the ammonium carbamate moieties not clearly be seen in Figure S5a, while they are clearly observed in Figure S5b. This is ascribed to a technical error arising from inhomogenities in the sample, rather than a slower formation rate of ammonium carbamate in the sample without CA.

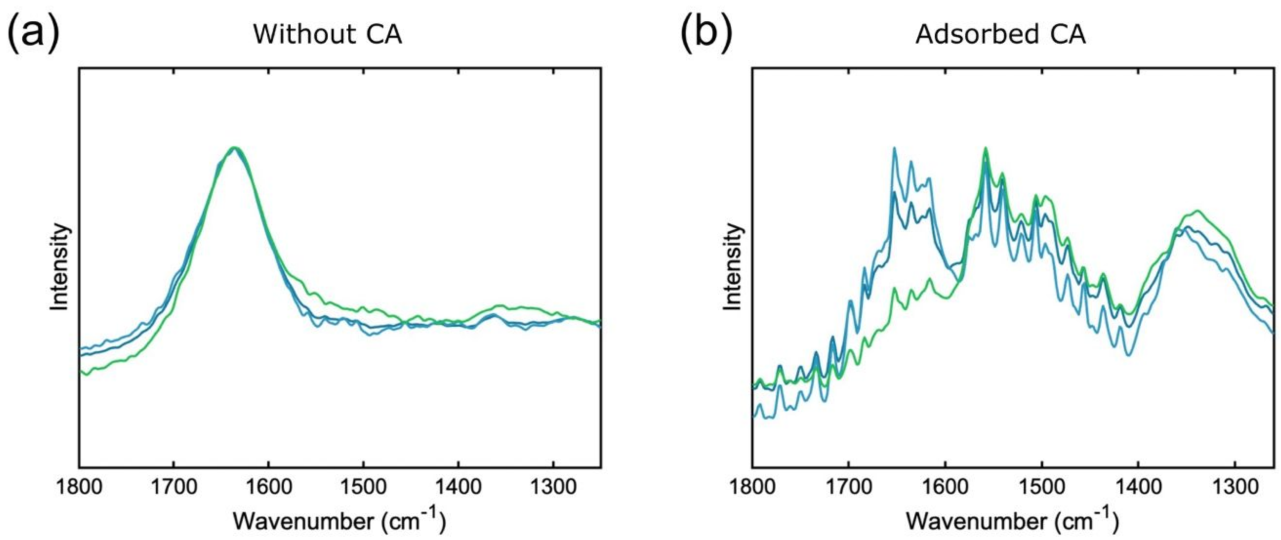


Figure S5. Supplementary IR spectra of aminated dispersion (a) without CA and (b) with adsorbed CA when exposed to $CO_2$.

## S5 Adsorption of hydrophobic dye ANS

The fluorescence spectra of adsorption of the hydrophobic dye 8-anilino-l-naphthalenesulfonic acid (ANS) to the free CA-s and immobilized CA-s are presented in Figure S6. The maximum intensity wavelengths are presented in Table S1.

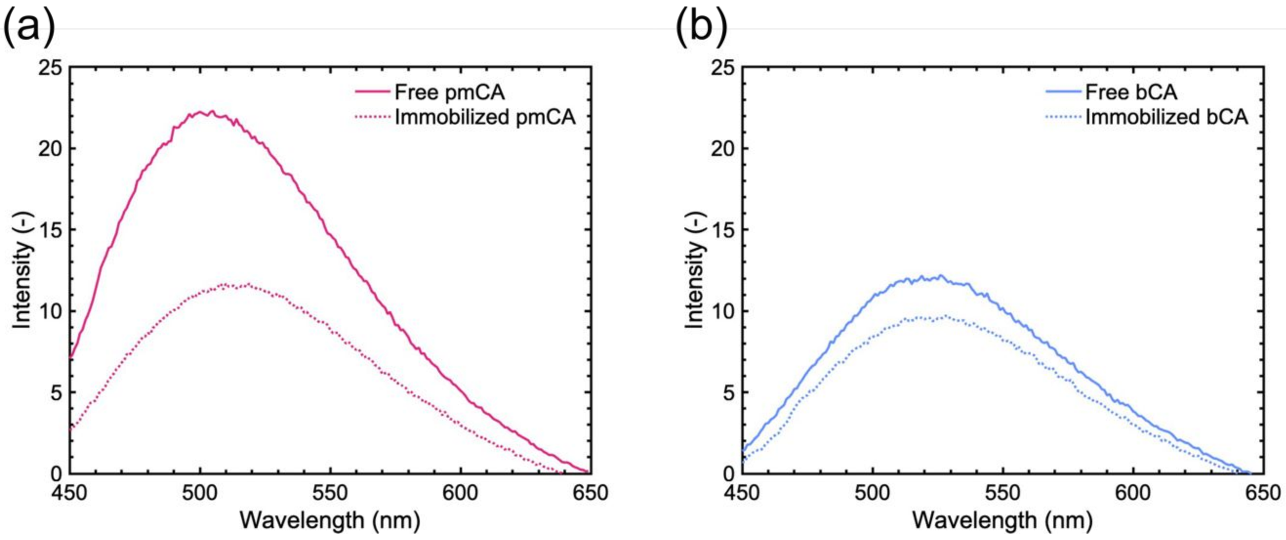


Figure S6. Fluorescence spectra of binding of the hydrophobic ANS to free and immobilized enzyme to (a) thermophilic pmCA and (b) commercial bovine bCA.

Table S2. Maximum intensity wavelength of fluorescence spectrum of free and immobilized thermophilic PM and commercial CA.

| | pmCA | bCA |
|---|---|---|
| Free | 505 nm | 526 nm |
| Immobilized | 529 nm | 529 nm |

## References


1. Sayari, A., Han, B.-H. & Yang, Y. Simple Synthesis Route to Monodispersed SBA-15 Silica Rods. *J. Am. Chem. Soc.* **126**, 14348–14349 (2004).

2. Zhao, D. et al. Triblock Copolymer Syntheses of Mesoporous Silica with Periodic 50 to 300 Angstrom Pores. Science 279, 548–552 (1998).

3. Wang, Y., Anyanwu, J.-T., Hu, Z. & Yang, R. T. Significantly enhancing CO2 adsorption on Amine-Grafted SBA-15 by boron doping and acid treatment for direct air capture. Separation and Purification Technology 309, 123030 (2023).

4. Yan, X., Zhang, L., Zhang, Y., Yang, G. & Yan, Z. Amine-Modified SBA-15: Effect of Pore Structure on the Performance for CO 2 Capture. Ind. Eng. Chem. Res. 50, 3220–3226 (2011).

5. Aziz, B., Zhao, G. & Hedin, N. Carbon Dioxide Sorbents with Propylamine Groups−Silica Functionalized with a Fractional Factorial Design Approach. Langmuir 27, 3822–3834 (2011).

6. Bacsik, Z. et al. Mechanisms and Kinetics for Sorption of CO 2 on Bicontinuous Mesoporous Silica Modified with n -Propylamine. Langmuir 27, 11118–11128 (2011).

7. Szego, A. E., Jaworski, A. & Hedin, N. Chemisorption of CO2 on diaminated silica as bicarbonates and different types of carbamate ammonium ion pairs. Mater. Adv. 2, 448–454 (2021).

8. Zhao, G., Aziz, B. & Hedin, N. Carbon dioxide adsorption on mesoporous silica surfaces containing amine-like motifs. Applied Energy 87, 2907–2913 (2010).

9. Polderman, L., Dillon, C. & Steele, A. Why monoethanolamine solution breaks down in gas-treating service. Oil Gas J 54, 180–183 (1955).
